# Associations between Bonus and Lottery COVID-19 Vaccine Incentive Policies and Increases in COVID-19 Vaccination Rates: A Social Epidemiologic Analysis

**DOI:** 10.3390/tropicalmed7070118

**Published:** 2022-06-26

**Authors:** Yuqi Guo, Jingjing Gao, Omar T. Sims

**Affiliations:** 1 School of Social Work, College of Health and Human Services, University of North Carolina at Charlotte, Charlotte, NC 28262, USA; yguo16@uncc.edu; 2 School of Data Science, University of North Carolina at Charlotte, Charlotte, NC 28262, USA; 3 Public Policy Program, University of North Carolina at Charlotte, Charlotte, NC 28262, USA; jgao9@uncc.edu; 4 Department of Health Behavior, School of Public Health, University of Alabama at Birmingham, Birmingham, AL 35222, USA; 5 Department of Social Work, College of Arts and Sciences, University of Alabama at Birmingham, Birmingham, AL 35222, USA; 6 Center for AIDS Research, School of Medicine, University of Alabama at Birmingham, Birmingham, AL 35222, USA; 7 Integrative Center for Aging Research, School of Medicine, University of Alabama at Birmingham, Birmingham, AL 35222, USA; 8 African American Studies, College of Arts and Sciences, University of Alabama at Birmingham, Birmingham, AL 35222, USA

**Keywords:** COVID-19, bonus and lottery incentive policies, COVID-19 vaccination rates, socioeconomic factors

## Abstract

The objectives of this longitudinal study were to analyze the impact of COVID-19 vaccine incentive policies (e.g., bonuses and lottery entries) on county-level COVID-19 vaccination rates, and to examine the interactive effects between COVID-19 vaccine incentive policies and socioeconomic factors on COVID-19 vaccination rates. Using publicly available data, county-level COVID-19 vaccination rates and socioeconomic data between January 2021 and July 2021 were extracted and analyzed across counties in the United States (US)—an analysis of 19,992 observations over time. Pooled ordinary least squares (OLS) analysis was employed to longitudinally examine associations with COVID-19 vaccination rates, and four random-effects models were developed to analyze interaction effects. Bonus incentive policies were effective in counties with a high per capita income, high levels of education, and a high percentage of racial minorities, but not in counties with high unemployment. Lottery incentive policies were effective in counties with a high percentage of racial minorities, but not in counties with high per capita income, high levels of education, and high unemployment. County-level socioeconomic factors should be considered ahead of implementing incentive policies, versus a blanket approach, to avoid the unintentional misuse of economic resources for futile COVID-19 vaccination outcomes.

## 1. Introduction

Between the start of the COVID-19 pandemic and April 2022, approximately 80 million COVID-19 cases and nearly 1 million associated deaths have been reported in the United States (US) [1]. COVID-19 vaccines are able to reduce the likelihood of infection and infection severity [2]. COVID-19 vaccine boosters are equally important as they provide the best protection against hospitalization and death, and against Coronavirus Omicron variants and foreseeable future variants [2,3,4,5]. As the outbreak expanded, COVID-19 vaccination emerged as the keystone of the government approach to curtail the spread of COVID-19 and protect the general population. Various public health policies were implemented at the state level across the US to improve COVID-19 vaccination rates, including but not limited to COVID-19 vaccination incentive policies [5,6].

The effectiveness of COVID-19 vaccination incentive policies remains debatable. Individual states have adopted multiple incentive approaches to incentivize and stimulate COVID-19 vaccination uptake in the general population, including bonus policies (e.g., guaranteed rewards and cash) and lottery policies (e.g., scholarships and cash). Some previous studies found that COVID-19 vaccine incentive programs were associated with an increase in COVID-19 vaccination rates in some states but not in others [7,8], while others reported that lottery policies effectively increased COVID-19 vaccination rates in many states [7,9,10]. For example, Ohio’s lottery policy—“Vax-a-Million”—led to around 50,000 to 100,000 additional first-dose COVID-19 vaccinations [7,11]. Vaccine incentives were found to induce some people who otherwise would not have been vaccinated to opt for vaccination or encourage people who would have been vaccinated to receive COVID-19 vaccines more quickly [11,12,13].

However, the impact of lottery policies on COVID-19 vaccination rates in Arkansas and California at the county level was not statistically significant [8]. A nationwide difference-in-difference analysis showed that there were no significant differences in COVID-19 vaccination trends between states with and without lottery incentive policies [14]. Small rewards (e.g., $5–50), bonuses, or low-probability lotteries may have been unable to combat and account for factors that may fuel COVID-19 vaccination hesitancy in unvaccinated individuals (e.g., misinformation and medical mistrust), especially if a considerable percentage (e.g., 40%) of individuals had already initiated COVID-19 vaccination by the time incentives were introduced [14].

Heterogeneous responses to vaccine incentive policies may be explained when accounting for socioeconomic factors. The ecological systems theory proposes that multi-level socioeconomic factors (microsystems, mesosystems, and macrosystems) impact health behaviors [15,16]. As such, it is plausible that socioeconomic factors may interact with the effects of incentive policies for COVID-19 vaccination. Microsystems refer to factors in the immediate environment, such as income, education, race, and age. Generally, income and education levels are positively associated with a willingness to vaccinate, as individuals with higher incomes and higher levels of education have better access to health care and greater vaccination literacy [17,18]. Compared to the White population, racial minorities have significantly lower vaccination rates for routinely recommended vaccines [19,20]. Mesosystems refer to indirect but prominent influential factors, such as community healthcare resources and local COVID-19 case rates. Clearly, the availability of COVID-19 vaccines, the number of COVID-19 cases, and related deaths are crucial indicators of vaccination rates [21]. Macrosystems refer to cultural influences, such as political beliefs and ideologies. Political ideology has been shown to significantly influence COVID-19 vaccination willingness [22,23].

Careful and purposeful consideration of the use of economic resources is required when implementing county and statewide incentive policies across the US. To inform future public health policies and to improve COVID-19 vaccination rates, rigorous examination and understanding of the factors associated with effective vaccination incentives are needed. To fill this knowledge and research gap, this nationwide longitudinal study aimed (1) to analyze the impact of COVID-19 vaccine incentive policies (i.e., lottery and bonus policies) on county-level COVID-19 vaccination rates, and (2) to examine interactive effects between COVID-19 vaccine incentive policies and socioeconomic factors on COVID-19 vaccination rates.

## 2. Materials and Methods

*Study Design.* This study conducted a longitudinal county-level analysis across states in the US, using secondary data from multiple sources: COVID-19 vaccination data from the US Centers for Disease Control and Prevention’s (CDC) COVID-19 Vaccine Tracker [1]; COVID-19 vaccine policies data from the National Government Association [24]; the support rate for Biden from the Pew Research Center [25]; and socioeconomic data (e.g., race, gender, age, education, and income) from the US Census Bureau [26]. The state of Texas was not included in the analysis because daily county-level COVID-19 data (e.g., numbers of vaccinations) were not provided for this state by the CDC’s COVID-19 Vaccine Tracker. Altogether, the study analyzed the longitudinal data from 2857 counties (19,992 observations) from January to July 2021.

*Dependent Variables.* The dependent variable was the daily COVID-19 vaccination rates in each US county.

*Independent Variables.* COVID-19 incentive policies were defined as policies that were specifically designed to motivate the public to receive COVID-19 vaccines, such as cash, food and entertainment vouchers, and bonuses. In measuring the various incentive policy programs, we coded the incentive policies into three categories: (1) no incentive policy; (2) bonus incentives, (i.e., food/entertainment vouchers or a small quantity of cash); and (3) lottery incentives (lottery drawings for cash and/or scholarships).

*Control Variables.* We controlled for the phasing of the COVID-19 vaccine distribution policy, according to the Vaccine Recommendations and Schedules of the CDC Advisory Committee on Immunization Practices (ACIP). These variables were assumed as: having followed the CDC ACIP vaccine recommendations and schedules (VRS) (defined as following the ACIP VRS); having expanded the eligibility of COVID-19 vaccination to more groups (e.g., young adults) more slowly than the VRS suggested was needed by the CDC ACIP (defined as slowly expanded eligibility); and having expanded the eligibility of COVID-19 vaccination to more groups (e.g., young adults) more quickly than the VRS suggested by the CDC ACIP (defined as quickly expanded eligibility) [27]. We also controlled for the number of days that the incentive policies were implemented in each county. Other control variables included the following for each county: support rate for President Biden, the percentage of Black, Indigenous, and people of color (BIPOC), the rates of adults with a bachelor’s degree, per capita income, the number of nurse practitioners, and the percentage of older adults (≥ 65 years old).

*Statistical Analysis*. Measures of central tendency and frequency distributions were used to characterize the study sample. Pooled ordinary least squares (OLS) analysis and a random-effects model were employed to longitudinally examine associations with COVID-19 vaccination rates. Four random-effects models were developed to analyze the following interaction effects on COVID-19 vaccination rates: (a) between county-level per capita income and incentive policies, (b) between county-level percentages of adults with a bachelor’s degree and incentive policies, (c) between county-level unemployment rates and incentive policies, and (d) between county-level percentages of BICOP and incentive policies.

## 3. Results

The descriptive results across 2857 counties over 7 time periods (i.e., January to July 2021) are provided in Table 1. The average county-level unemployment rate was 6.71 (SD = 2.23), the average per capita income at the county level was 25,074.69 (SD = 5999.78), and the average percentage of adults with a bachelor’s degree at the county level was 21.82 (9.55); the mean percentage of BIPOC across counties was 0.15% (SD = 0.16), and the average county-level number of nurse practitioners was 54.29 (SD = 1545.66).

The dependent variable (rates of COVID-19 vaccination at the county level) and some independent variables (e.g., the number of days that COVID-19 vaccines were available to the general population, or the number of days of implementation of the incentive policies) varied over time and across counties. Table 2 provides information on within and between variations. The within variation measures variations over time in an individual county, while the between variation represents variations over time across all included counties. The within variation of the rate of COVID-19 vaccination was 13.97, and the between variation of the rate of COVID-19 vaccination was 6.00.

### 3.1. Associations between COVID-19 Vaccine Incentive Policies and County-Level Vaccination Rates

The first aim of our study was to evaluate the relationship between COVID-19 incentive policies and COVID-19 vaccination rates. Table 3 presents the results of the pooled OLS and random-effects models. In the pooled OLS model, incentive policies were positively associated with COVID-19 vaccination rates. Compared to counties without any incentive policies, counties with bonus policies had a 223.0% increase in COVID-19 vaccination rates, while counties with lottery policies had a 134.3% increase in COVID-19 vaccination rates.

Regarding socioeconomic factors, the county-level per capita income (*p* < 0.001), the number of nurse practitioners (*p* < 0.001), the percentage of adults aged 65 and over (*p* < 0.001), and political support for Biden (*p* < 0.001) were positively associated with COVID-19 vaccination rates at the county level; however, the county level percentage of BIPOC (*p* < 0.001) was negatively associated with COVID-19 vaccination rates at the county level.

### 3.2. Interaction Effects

**Figure 1:**
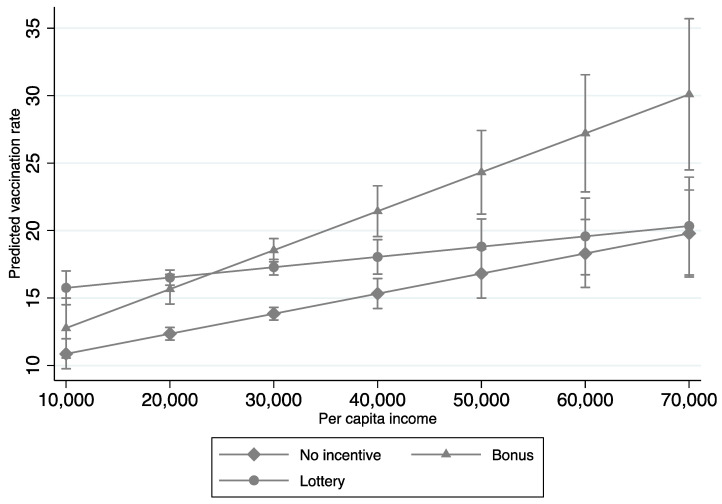
Interaction effects of COVID-19 vaccination incentive policies and per capita income (Model 3 in Table 3).

**Figure 2:**
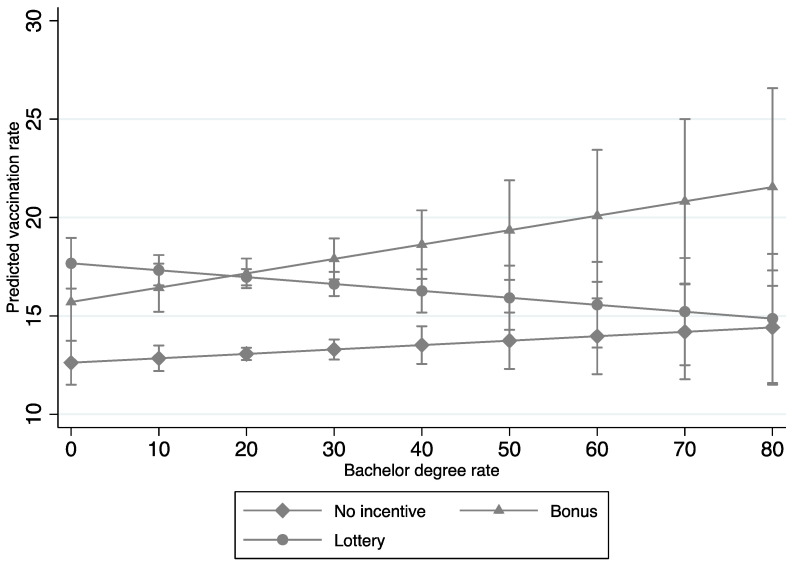
Interaction effects of COVID-19 vaccination incentive policies and rates of adults with bachelor’s degrees (Model 4 in Table 3).

**Figure 3:**
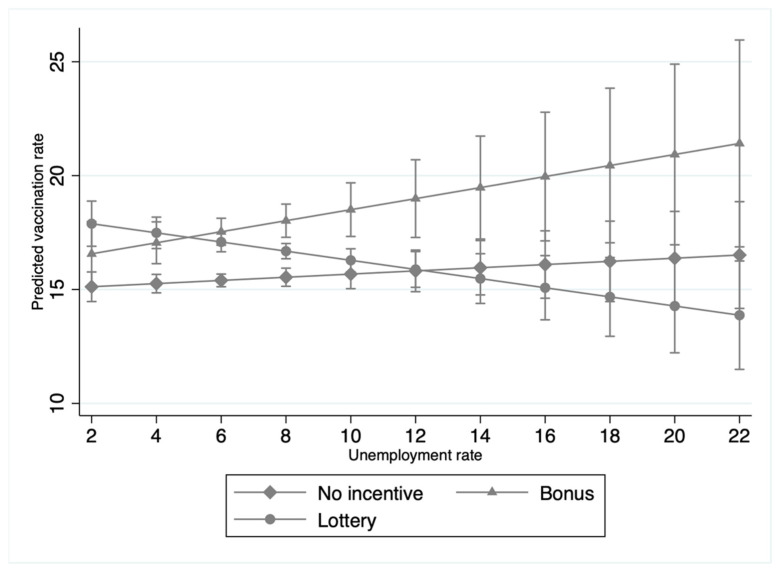
Interaction effects of COVID-19 vaccination incentive policies and unemployment rates (Model 5 in Table 3).

**Figure 4:**
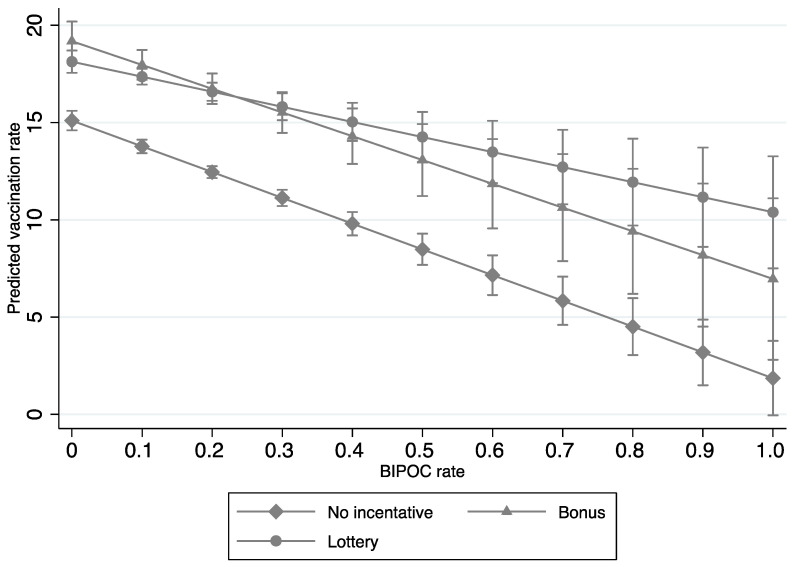
Interaction effects of COVID-19 vaccination incentive policies and rates of BIPOC (Model 6 in Table 3).

The second aim of our study was to analyze the interaction effects between socioeconomic factors (e.g., per capita income, education, unemployment, and race) and incentive policies on COVID-19 vaccination rates. Significant interaction effects were found between bonus policies and per capita income, education, and race, and between lottery policies, unemployment, and race (Table 3); these respective interaction effects are graphed in Figure 1, Figure 2, Figure 3 and Figure 4. In terms of counties with bonus policies, those counties with higher county-level per capita income (Figure 1), higher county-level rates of adults with a bachelor’s degree (Figure 2), and higher county-level percentages of BIPOC (Figure 3) were positively associated with increases in COVID-19 vaccination rates. In terms of counties with lottery policies, those counties with higher county-level percentages of BIPOC were positively associated with increases in COVID-19 vaccination rates (Figure 4), but those with higher county-level unemployment rates were negatively associated with COVID-19 vaccination rates (Figure 3).

## 4. Discussion

This study analyzed the impact of lottery policies and bonus policies on COVID-19 vaccination rates and examined the interactive effects of lottery incentive policies and bonus incentive policies with several socioeconomic factors regarding county-level COVID-19 vaccination rates. Several main findings materialized from the study. First, bonus and lottery incentive policies for COVID-19 vaccination rates significantly increased COVID-19 vaccination rates but, interestingly, bonus incentive policies increased COVID-19 vaccination rates at a far greater level than lottery incentive policies. Our findings of incentive policies increasing COVID-19 vaccination rates are consistent with those in previous studies [11,12,13,14,15]. Fogg’s behavior model (FBM) [28] provides insight into why this association—the positive relationship between incentive programs and health behavior change—is likely to occur. COVID-19 vaccines have garnered widespread public skepticism since being approved by the US Food and Drug Administration, and many people feel ambivalent regarding COVID-19 vaccination [14]. However, the monetary component of monetary-based incentive programs is likely to trigger consideration of and motivation for COVID-19 vaccination uptake. This appeals to the psychological aspects of health behavior change, wherein a new motivation is introduced to further incentivize willingness and action [29]. This is of particular importance, and it is perhaps ideal and most successful with vaccine-hesitant individuals who may be stimulated to uptake COVID-19 vaccination via monetary-based incentives [29].

In general, county-level socioeconomic factors moderated the effects of bonus and lottery incentive policies on COVID-19 vaccination rates. In particular, our findings suggest that bonus and lottery incentive policies work differently in counties with different socioeconomic statuses. The second and third main findings of this study are that in counties implementing bonus incentive policies, COVID-19 vaccination rates were associated with increases in counties with higher per capita income and education, but lottery incentive policies had no significant impact in the context of higher per capita income and education; while in counties with lottery incentive policies, COVID-19 vaccination rates were associated with decreases in those counties with high unemployment. Altogether, this suggests that public-health COVID-19 vaccination campaigns may want to consider the use of bonus incentive policies in counties with higher per capita income and a greater percentage of adults with a bachelor’s degree, along with the avoidance of lottery incentive policies in counties with higher unemployment rates.

The fourth finding is that both bonus and lottery incentive policies were associated with increased COVID-19 vaccination rates in counties with a higher percentage of BIPOC; in particular, lottery incentive policies were the most optimal. The majority of published studies within the context of lottery-based incentives and COVID-19 vaccination rates present mixed findings. However, the majority of those respective studies were cross-sectional, were conducted in a single state (e.g., Ohio), or did not examine the interactive effects with race [11,13,14,30,31,32]. The current findings from our longitudinal study across the US at the county level suggest that policymakers and public health officials may want to give strong consideration to the use of lottery incentive policies in counties with a higher percentage of BIPOC, to upturn COVID-19 vaccination rates.

This study had noteworthy strengths and limitations. The longitudinal and national scope of the analysis considerably strengthened the study’s external validity, as longitudinal data over consecutive time points from more than 90% of counties in the US were included in the analysis. However, Texas was not included in the analysis as the daily county-level COVID-19 data for Texas was not provided by the CDC’s COVID-19 Vaccine Tracker. As such, the findings cannot be generalized to Texas. Future studies may want to consider the use of other data sources to include the state of Texas in iterative analyses. Inferences regarding causality cannot be inferred. The analysis was limited to lottery and bonus incentive policies. Future studies are also encouraged to examine other incentive policies.

## 5. Conclusions

Public health policymakers are encouraged to be mindful that the effects of lottery incentive and bonus incentive policies vary in the US, according to county-level socioeconomic factors. Although bonus incentives appear to be the most effective, county-level socioeconomic factors should be considered when designing and implementing targeted incentive policies, versus applying a blanket approach, to avoid the unintentional waste of economic resources on futile COVID-19 vaccination uptake outcomes because lottery-based incentive policies may be more optimal, depending on a county’s socioeconomic profile. Public health and policy efforts that are guided by empirical evidence are more likely to improve COVID-19 vaccination uptake at the population level and to make good use of economic resources.

## Figures and Tables

**Table 1:** Descriptive statistics of COVID-19 vaccination incentive policies and socioeconomic factors at the county level.

Variable	Frequency (*n*)	Percentage (%)	Mean	Std. Dev.	Min	Max
Panel time range					1 Jan 2021	1 Jul 2021
Rate of vaccinated population per county			16.14	15.20	0.00	99.90
Incentive policies:						
No incentives	10,367	51.84				
Bonus incentives	2233	11.17				
Lottery incentives	7399	37.00				
Number of days of implementation of incentive policies			2.46	8.90	0.00	55.00
ACIP VRS Phasing:						
Followed the ACIP VRS	13,972	64.16				
Slowly expanded the ACIP VRS	448	2.06				
Quickly expanded the ACIP VRS	7357	33.78				
Number of days that COVID-19 vaccines were available to the general population			25.07	33.18	0.00	107.00
Biden support rate			0.34	0.16	0.05	0.92.00
Number of nurse practitioners			54.29	15,45.66	0.23	3937.77
Unemployment rate			6.71	2.23	1.70	22.50
Per capita income			25,074.69	5999.78	9688.43	66,518.36
Percentage of adults with a bachelor’s degree			21.82	9.55	5.40	78.50
Rate of BIPOC			0.15	0.16	0.01	0.94
Percentage of people aged 65 and above			0.19	0.05	0.00	0.58

Note: ACIP = CDC Advisory Committee on Immunization Practices; VRS = vaccine recommendations and schedules; BIPOC = Black, Indigenous, and people of color.

**Table 2:** Within and between variations of COVID-19 vaccination policies’ panel data.

Variables		Mean	Std. Dev.	Min	Max	Observations
Rate of COVID-19 vaccination	overall	16.14	15.20	0.00	99.90	N = 19,999
	between		6.00	0.00	61.14	*n* = 2857
	within		13.97	−45.01	62.94	T = 7
Number of days that COVID-19 vaccines were available to the general population	overall	25.07	33.18	0.00	107.00	N = 19,999
	between		3.57	18.29	35.14	*n* = 2857
	within		32.99	−10.07	96.93	T = 7
Number of days of implementation of incentive policies	overall	2.46	8.90	0.00	55.00	N = 19,999
	between		3.45	0.00	11.43	*n* = 2857
	within		8.20	−8.97	46.03	T = 7
ACIP VRS phasing	overall	0.76	0.96	0.00	2.00	N = 19,999
	between		0.96	0.00	2.00	*n* = 2857
	within		0	0.76	0.76	T = 7
Biden support rate	overall	0.34	0.16	0.05	0.92	N = 19,999
	between		0.16	0.05	0.92	*n* = 2857
	within		0.00	0.34	0.34	T = 7
Number of nurse practitioners	overall	54.29	155.66	0.23	3937.77	N = 19,999
	between		155.68	0.23	3937.77	*n* = 2857
	within		0.00	54.29	54.29	T = 7
Unemployment rate	overall	6.71	2.23	1.70	22.5	N = 19,999
	between		2.23	1.70	22.5	*n* = 2857
	within		0.00	6.71	6.71	T = 7
Per capita income	overall	25,074.69	5999.80	9688.43	66,518.36	N = 19,999
	between		6000.70	9688.43	66,518.36	*n* = 2857
	within		0.00	25,074.69	25,074.69	T = 7
Percentage of adults with a bachelor’s degree	overall	21.82	9.55	5.40	78.50	N = 19,999
	between		9.55	5.40	78.50	*n* = 2857
	within		0.00	21.82	21.81	T = 7
Rate of BIPOC populations	overall	0.16	0.16	0.01	0.94	N = 19,992
	between		0.16	0.01	0.94	*n* = 2856
	within		0.00	0.16	0.15	T = 7
Percentage of people aged 65 and above	overall	0.19	0.05	0.00	0.58	N = 19,999
	between		0.05	0.00	0.58	*n* = 2857
	within		0.00	0.19	0.19	T = 7

Note: N represents the total observations in the panel data; *n* indicates the total number of counties in the panel data; T is the total number of periods in this panel data. The dataset is of panel data, and the dependent variable (rate of vaccinated population per county) and key independent variables (the number of days the vaccination was offered to the general population and of incentive policies) vary over both time and individual county. Within variation means the variation over time, given an individual county (if the within variation equals 0, it means that this variable is not time-sensitive in this study period); between variation represents variation across counties; overall variation is the variation over time and across counties. ACIP = CDC Advisory Committee on Immunization Practices; VRS = vaccine recommendations and schedules; BIPOC = Black, Indigenous, and people of color.

**Table 3:** Time series analysis of COVID-19 vaccination incentive policies and interaction effects between COVID-19 vaccination incentive policies and socioeconomic factors.

	Random Effects					
Variables (DV = County Level COVID-19 Vaccination Rate)	(1) Pooled OLS	(2) Initial	(3) Per Capita Income	(4) Percentage of Adults with a Bachelor’s Degree	(5) Unemployment Rate 2020	(6) Percentage of BIPOC
Bonus incentive policies	2.230 ***	2.281 ***	−0.798	0.764	1.101	1.641 ***
Lottery incentive policies	1.343 ***	1.376 ***	1.497	1.284 *	3.310 ***	−0.116
Slowly expanded the ACIP VRS	0.324	0.300	0.257	0.298	0.349	−1.385
Quickly expanded the ACIP VRS	−1.084 ***	−1.078 ***	−1.068 ***	−1.062 ***	−1.168 ***	−0.986 ***
Number of nurse practitioners	0.00279 ***	0.00279 ***	0.00277 ***	0.00276 ***	0.00297 ***	0.00196 **
Unemployment rates	−0.0160	−0.0169	−0.0287	−0.0245	0.0696	0.0251
Per capita income	0.000159 ***	0.000159 ***	0.000146 ***	0.000156 ***	0.000159 ***	0.000144 ***
Percentage of adults with bachelor’s degrees	−0.0153	−0.0155	−0.0154	−0.0247	−0.0165	−0.0121
Rate of BIPOC	−15.04 ***	−15.04 ***	−15.01 ***	−15.13 ***	−15.38 ***	−18.23 ***
Percentage of people aged 65 and above	17.73 ***	17.73 ***	17.87 ***	17.81 ***	17.60 ***	16.89 ***
Biden support rate	19.66 ***	19.67 ***	19.71 ***	19.79 ***	19.59 ***	19.63 ***
Bonus × Per capita income			0.000120 *			
Lottery × Per capita income			−0.0000001			
Bonus × Percentage of adults with a bachelor’s degree				0.0691 *		
Lottery × Percentage of adults with a bachelor’s degree				0.00465		
Bonus × Unemployment rates					0.173	
Lottery × Unemployment rates					−0.270 *	
Bonus × Rate of BIPOC populations						4.168 *
Lottery × Rate of BIPOC populations						10.44 ***
Constant	−4.363 ***	−4.418 ***	−4.063 ***	−4.147 ***	−4.790 ***	−3.663 ***
Observations	19,992	19,992	19,992	19,992	19,992	19,992
R^2^	0.604					
R^2^—within		0.667	0.667	0.667	0.667	0.667
R^2^—between		0.261	0.263	0.262	0.264	0.273
R^2^—overall		0.604	0.604	0.604	0.604	0.606
Number of counties		2856	2856	2856	2856	2856

Note: *** *p* < 0.001, ** *p* < 0.01, * *p* < 0.05. ACIP = CDC Advisory Committee on Immunization Practices; VRS = vaccine recommendations and schedules; BIPOC = Black, Indigenous, and people of color.

## Data Availability

This study analyzed publicly available datasets. These datasets can be found here: https://www.census.gov/programs-surveys/ces/data/restricted-use-data/demographic-data.html and here: https://data.cdc.gov/Vaccinations/COVID-19-Vaccinations-in-the-United-States-County/8xkx-amqh (accessed on 21 August 2021).
